# Four *Escherichia coli* O157:H7 Phages: A New Bacteriophage Genus and Taxonomic Classification of T1-Like Phages

**DOI:** 10.1371/journal.pone.0100426

**Published:** 2014-06-25

**Authors:** Yan D. Niu, Tim A. McAllister, John H. E. Nash, Andrew M. Kropinski, Kim Stanford

**Affiliations:** 1 Alberta Agriculture and Rural Development, Agriculture Centre, Lethbridge, Alberta, Canada; 2 Lethbridge Research Centre, Agriculture and Agri-Food Canada, Lethbridge, Alberta, Canada; 3 Public Health Agency of Canada, Laboratory for Foodborne Zoonoses, Guelph, Ontario, Canada; 4 Department of Pathobiology, Ontario Veterinary College, University of Guelph, Guelph, Ontario, Canada; 5 Department of Cellular and Molecular Biology, University of Guelph, Guelph, Ontario, Canada; Centro Nacional de Biotecnologia – CSIC, Spain

## Abstract

The T1-like bacteriophages vB_EcoS_AHP24, AHS24, AHP42 and AKS96 of the family *Siphoviridae* were shown to lyse common phage types of Shiga toxin-producing *Escherichia coli* O157:H7 (STEC O157:H7), but not non-O157 *E. coli*. All contained circularly permuted genomes of 45.7–46.8 kb (43.8–44 mol% G+C) encoding 74–81 open reading frames and 1 arginyl-tRNA. Sodium dodecyl sulfate-polyacrylamide gel electrophoresis revealed that the structural proteins were identical among the four phages. Further proteomic analysis identified seven structural proteins responsible for tail fiber, tail tape measure protein, major capsid, portal protein as well as major and minor tail proteins. Bioinformatic analyses on the proteins revealed that genomes of AHP24, AHS24, AHP42 and AKS96 did not encode for bacterial virulence factors, integration-related proteins or antibiotic resistance determinants. All four phages were highly lytic to STEC O157:H7 with considerable potential as biocontrol agents. Comparative genomic, proteomic and phylogenetic analysis suggested that the four phages along with 17 T1-like phage genomes from database of National Center for Biotechnology Information (NCBI) can be assigned into a proposed subfamily “Tunavirinae” with further classification into five genera, namely “Tlslikevirus” (TLS, FSL SP-126), “Kp36likevirus” (KP36, F20), *Tunalikevirus* (T1, ADB-2 and Shf1), “Rtplikevirus” (RTP, vB_EcoS_ACG-M12) and “Jk06likevirus” (JK06, vB_EcoS_Rogue1, AHP24, AHS24, AHP42, AKS96, phiJLA23, phiKP26, phiEB49). The fact that the viruses related to JK06 have been isolated independently in Israel (JK06) (GenBank Assession #, NC_007291), Canada (vB_EcoS_Rogue1, AHP24, AHS24, AHP42, AKS96) and Mexico (phiKP26, phiJLA23) (between 2005 and 2011) indicates that these similar phages are widely distributed, and that horizontal gene transfer does not always prevent the characterization of bacteriophage evolution. With this new scheme, any new discovered phages with same type can be more properly identified. Genomic- and proteomic- based taxonomic classification of phages would facilitate better understanding phages diversity and genetic traits involved in phage evolution.

## Introduction

Tailed bacteriophages (phages) with double-strand DNA genomes belonging to the order *Caudovirales* are the most abundant viruses on earth, accounting for 96% of all the phages observed [1]. Based on tail morphology, these viruses are classified by the International Committee on Taxonomy of Viruses (ICTV), into three families−*Myoviridae* (long contractile tail), *Siphoviridae* (long non-contractile tail) and *Podoviridae* (short non-contractile tail). Recent advances in sequencing technologies has led to a proliferation in the sequencing of phage genomes [2], [3], enabling comparative genomics and proteomics to better define phage taxonomy. The family *Myoviridae* now contains three subfamilies, *Peduovirinae*, *Spounavirinae* and *Tevenvirinae* [4], and 18 genera. The family *Podoviridae* has been further divided into the *Autographivirinae and Picovirinae* subfamilies [2], with a total of eleven genera. The *Siphoviridae* account for >61% of described phages [1] and this family also represents the largest group of fully sequenced phages, but no subfamilies, and only nine bacterial-specific phage genera have been described. Classification of the *Siphoviridae* is currently under review by ICTV (Adriaenssens, personal communication). T1-like phages possess terminally redundant and circularly permuted genomes of ∼50 kb, and are currently classified as members of one genus (*Tunalikevirus*) within *Siphoviridae* [5]. Morphologically, they have a polyhedral head 60 nm in diameter with an extremely flexible non-contractile tail 151 nm in length and 8 nm in diameter [6]. At present, ICTV only recognizes nine species of phages within this genus with 1, 1, 6 and 1 infecting *Cronobacter, Enterobacter, Escherichia coli*, and *Shigella*, respectively.

Shiga-toxin producing *E. coli* O157:H7 (STEC O157:H7) remains one of leading causes of foodborne illnesses in North America [7], [8]. Although the food production continuum has introduced control measures to prevent the pathogen from entering food chain, outbreaks of STEC O157:H7 linked to fresh produce and beef products continue (http://www.cdc.gov/ecoli/outbreaks.html and http://www.phac-aspc.gc.ca/fs-sa/fs-fi/ecoli-eng.php). Lytic phages offer promise in the prevention and therapy of bacterial infections in humans [9], livestock [10], [11] and plants [12] and have been employed to decontaminate processed foods and agricultural products [9], [10]. However, the use of phage therapy to target bacterial pathogens such as STEC O157:H7 [13], [14] and *Salmonella* [15], [16] in the digestive tract of livestock remains challenging. Factors such as the development of phage resistance, the complexity of predator-prey relationships between phages and hosts, the diversity and abundance of microflora in the gastro-intestinal tract all may undermine the effectiveness of phage therapy. Recently, *in-vitro* experiments in our laboratory have indicated that competitive interference between different phage types may be another factor impacting effectiveness of phage cocktails [17], even though this approach is often advocated as a means of avoiding resistance. An improved understanding of phage taxonomy, proteomics and target receptors could lead to the formulation of more effective phage cocktails that overcome resistance development while remaining efficacious.

Previously, four STEC O157:H7-infecting bacteriophages (vB_EcoS_AHP24, AHS24, AHP42 and AKS96) originally isolated from cattle feedlots in southern Alberta, Canada were classified as T1-like *Siphoviridae* by electron microscopy, but exhibited divergent genotypes based on EcoRI- or HindIII-digestion profiles [18]. This study aimed to further define their genomic and proteomic characteristics as well as infectivity against STEC O157:H7 and non-pathogenic *E. coli* (ECOR) strains. We also conducted comparative genomic, proteomic and phylogenetic analysis among known T1-like phages in an effort to determine how these viruses could be optimally classified.

## Materials and Methods

### Bacteriophage, bacteria and media

Four phages infecting STEC O157:H7 strain R508 (phage type, PT14) were isolated from the feces of commercial feedlot cattle in 2007 in Alberta, Canada [19] with AHP24 (Pen 10), AHS24 (Pen 10) and AHP42 (Pen 6) from Feedlot B and AKS97 (Pen 2) from Feedlot A [19] with permission. A single discrete plaque from each phage was purified three times by the soft agar (0.6%) overlay method [20] and propagated as previously discribed [18]. Titers of phages in the stock filtrates were then determined by the soft agar overlay technique [20]. STEC O157:H7 strain R508 was used as a host for plaque purification, propagation and titration of the phage stocks. Other standard laboratory strains of STEC O157:H7 (n = 24) and non-O157 *E. coli* (n = 73) [21] used to evaluate host range of four T1-like phages are listed in Table 1. Unless otherwise indicated the bacterial strains were grown in tryptic soy broth and/or tryptic soy agar.

**Table 1:** Host range and lytic capability of phages AHP24, AHS24, AHP42 and AKS96.

Bacteria	Strains^a^	Sensitivity^b^ of T1-like phages			
		AHP24	AHS24	AHP42	AKS96
STEC O157:H7	PT8, 33, 38	+	+	+	+
	PT10, 14a, 28, 32, 34, 46−48, 54, 68, 80, 88	+++	+++	+++	+++
	PT24	+++	+++	+++	+
	PT31	++	++	++	++
	PT45	+++	+++	++	+++
	PT49	++	++	++	++
	PT50, 67	+++	+++	++	++
	PT51	+++	+++	+	+
	PT63	++	++	+	+
	PT74	++	++	+++	+++
non-O157 *E. coli*	ECOR collection^c^	−	−	−	−

a PT represents phage type of STEC O157:H7 strains

b Sensitivities are grouped on the basis of multiplicity of infection (MOI: the lowest ratio of phage to bacteria that resulted in complete lysis of an overnight bacterial culture during 5 h of incubation with serial dilutions of the phage). +++: extremely susceptible (MOI < 0.01); ++: highly susceptible (0.01 ≤ MOI <1); +: moderately susceptible (1 ≤ MOI < 10);

–: non-susceptible (i.e., no lysis observed).

c ECOR collection represents standard reference strains of *Escherichia coli* [21].

### Host range and lytic capability

Host range and lytic capability of the phages for STEC O157:H7 and non-O157 *E. coli* was assessed using a microplate phage virulence assay [22]. To estimate multiplicity of infection (MOI), high titre phage stocks (10^9^−10^10^ PFU/ml) were serially diluted and incubated at 37°C for 5 h with 10-fold diluted overnight cultures of STEC O157:H7 in a 96-well microplate. After incubation, wells were examined visually for turbidity and the highest dilution that resulted in complete lysis (no discernable turbidity) of bacteria was recorded. The MOI for each phage-host assay was calculated by dividing the initial number of phages in the highest-dilution wells by the initial number of bacteria added, as determined by plate counts of serially diluted bacterial cultures.

### CsCl density gradient centrifugation

Bacterial nucleic acids were removed from filtered phage lysates (∼10^9^ PFU/ml) using DNase1 (Sigma-Aldrich, Oakville, ON, Canada) and RNaseA (Sigma-Aldrich), and the phage lysates were concentrated in polyethylene glycol (PEG) 8000 and purified through two rounds of CsCl density gradient centrifugation [20].

### Genome sequencing and annotation

Phage DNA was extracted from the CsCl-purified phage lysates using the SDS-proteinase K protocol of Sambrook and Russell [20]. Purified phage DNA was submitted to the Plate-forme d'Analyses Génomiques of the Institut de Biologie Intégrative et des Systèmes (Laval University, Québec, QC, Canada) for sequencing. For each sample, a tagged GS-FLX rapid library was made according to the manufacturer's instructions (Roche/454 sequencing, Brandford, USA). Phage libraries were pooled for sequencing on a GS-FLX+ instrument using titanium chemistry according to the manufacturer's instructions (Roche/454). Sequencing reads were assembled with the gsAssembler module of Newbler v. 2.5.3.

Initial genome annotation was completed using myRAST [23]. Geneious v5.4 program (Biomatters Ltd., Auckland, New Zealand) was used to visually scan the sequence for potential genes. All predicted proteins were scanned for homologues using BLASTP and PSI-BLAST [24]. Rho-independent terminators were identified using WebGeSTer at http://pallab.serc.iisc.ernet.in/gester/rungester.html [25] and TransTermHP [26]. Promoters were identified by neural network promoter prediction [27] with visual inspection. Transfer RNA (tRNA) genes were screened using Aragorn [28] at http://130.235.46.10/ARAGORN/and tRNAScan at http://lowelab.ucsc.edu/tRNAscan-SE/ [29]. Transmembrane domains were described using TMHMM 2.0 at http://www.cbs.dtu.dk/services/TMHMM/ [30], Phobius at http://phobius.sbc.su.se/ [31] and SPLIT 4.0 at http://split.pmfst.hr/split/4/ [32]. The phage genomes were rendered syntenic by opening at the initiation codon for the small subunit terminase gene prior to dotplot alignment, ClustalW alignment and EMBOSS Stretcher analysis. Whole genome sequences of the four phages studied, as well as another 17 T1-like phages (Table 2) from the database of National Center for Biotechnology Information (NCBI) were analyzed by local ClustalW algorithm [33], [34] using default parameters and phylogenetic trees were visualized by FigTree program (available from http://tree.bio.ed.ac.uk/software/figtree/). CLUSTAL omega [35] was used to align amino acid sequences of T1-like phages. The GenBank accession numbers for AHP24, AHS24, AHP42 and AKS96 sequence are KF771236, KF771238, KF771237 and KF771239, respectively.

**Table 2:** Features of the T1-like phages from NCBI database.

Phages	Phylogenic Cluster	Host	Head Dimension (nm)	Tail Dimension (nm)	Genome size (bp)	Mole% G+C	Reference	Accession #
TLS	A	*E. coli*, *Shigella*	NA	NA	49,902	42.7	51	NC_009540
FSL SP-126	A	*Salmonella*	NA	NA	51,092	42.9	53	KC139513
vB_KpnS_KP36	B	*Klebsiella*	NA	NA	49,818	50.7	55	NC_019781
F20	B	*Enterobacter*	50	150×7	51,543	47.9	54	JN672684
T1	C	*E. coli*, *Shigella*	60	151×8	48,836	45.6	51	NC_005833
Shfl1	C	*Shigella*	NA	NA	50,661	45.4	NA	NC_015456
ADB-2	C	*E. coli*	NA	NA	50,552	46	52	NC_019725
RTP	D	*E. coli*	NA	NA	46,219	44.3	57	NC_007603
vB_EcoS_ACG-M12	D	*E. coli*	57	172×7	46,054	44	56	NC_019404
phiEB49	E	*E. coli*	50	NA	47,180	44	58	JF770475
vB_EcoS_Rogue1	E	*E. coli*	53	152×8	45,805	44.2	40	NC_019718
phiJLA23	E	*E. coli*	NA	NA	43,017	44.2	49	KC333879
phiKP26	E	*E. coli*, *Salmonella*	NA	NA	47,285	44.3	50	KC579452
JK06	E	*E. coli*	NA	NA	46,072	44	NA	NC_007291
pSf-1	NA	*Shigella*	73	103×13	51,821	44	59	NC_021331
ESP2949-1	NA	*Cronobacter*	NA	NA	49,116	50.1	60	NC_019509
vB_XveM_DIBBI	NA	*Xanthomonas*	NA	NA	49,981	52.4	NA	NC_017981

a NA: Not applicable.

### Analysis of structural proteins

The in-gel digest and mass spectrometry experiments were performed by the Proteomics platform of the Eastern Quebec Genomics Center (Quebec, Canada). CsCl-purified phage particles were analyzed for structural proteins by standard Tris-glycine 12% sodium dodecyl sulfate-polyacrylamide gel electrophoresis (SDS-PAGE). Samples were mixed with sample loading buffer and boiled for 5 min before loading. Proteins were stained with Coomassie brilliant blue R250 (Bio-Rad Laboratories, Mississauga, ON, Canada) and subsequently characterized using Bionumerics 6.6 software (Applied Maths, Austin, TX, USA). Bands of interest were excised and de-stained with water. Tryptic digestion was performed on a MassPrep liquid handling robot (Waters, Milford, USA) according to the manufacturer's specifications and to the modified protocol [36], [37]. Briefly, following reduction with 10 mM dithiothreitol (DTT) and alkylation with 55 mM iodoacetamide, the protein was digested by 126 nM of modified porcine trypsin (Sequencing grade, Promega, Madison, WI, USA) at 58°C for 1 h. The proteolytic peptides were then extracted using 1% formic acid, 2% acetonitrile followed by 1% formic acid and 50% acetonitrile. The recovered extracts were pooled, dried by vacuum centrifuge and resuspended into 7 µl of 0.1% formic acid for mass spectrometry. Peptide resuspensions (2 µl) were separated by online reversed-phase (RP) nanoscale capillary liquid chromatography (nanoLC) and analyzed by electrospray mass spectrometry (ES MS/MS). The experiments were performed with a Agilent 1200 nano pump connected to a triple time-of-flight mass spectrometer (AB Sciex 5600, Framingham, MA,USA) equipped with a nanoelectrospray ion source (AB Sciex 5600). Briefly, 2 µl of the peptide resuspension was injected onto a 15 cm×75 µm (internal diameter) PicoFrit column (New Objective, Woburn, MA), packed with reversed phase C18 particles (5 µm diameter; 300 Å pore size; Jupiter 300, Phenomenex, Torrance, CA, USA) and eluted in a linear gradient from 2–50% buffer B (0.1% formic acid in acetonitrile) at flow rate of 300 nl/min for 30 min. Mass spectra were acquired using a data-dependent acquisition mode using Analyst software version 1.6 (AB Sciex 5600). Each full scan mass spectrum (400 to 1250 m/z) was followed by collision-induced dissociation of the twenty most intense ions. Dynamic exclusion was set for a period of 3 sec and a tolerance of 100 ppm.

All MS/MS peak lists were generated with ProteinPilot Version 4.5 (AB Sciex, Framingham, MA, USA) and analyzed using Mascot (Matrix Science, London, UK; version 2.3.02). Proteins were identified by searching against the Uniref100-SiphoViridea database (release 12-05) as well as an in-house protein database derived from genome sequences of AHP24, AHS24, AHP42 and AKS96. Parameters for Mascot used a fragment ion mass tolerance of 0.10 Da and a parent ion tolerance of 0.10 Da. Iodoacetamide derivatives of cysteine were specified as a fixed modification and oxidation products of methionine were specified as a variable modification with up to two missed cleavages allowed. Scaffold version 4.0.1 (Proteome Software Inc., Portland, OR, USA) was used to validate MS/MS based peptide and protein identifications. The protein identification cut off was set at a confidence level of 95% (MASCOT score >33) with a requirement for at least two peptides to match to a protein. Proteins with similar peptides that could not be differentiated based on MS/MS analysis alone were grouped to satisfy the principles of parsimony.

## Results

Phages were able to lyse all 24 STEC O157:H7 strains tested, but displayed no activity against any of the 73 non-O157 *E. coli* strains (Table 1). Phages AHP24 and AHS24 exhibited the same infective pattern, with 17 strains extremely susceptible, 4 strains highly susceptible and 3 strains moderately susceptible. On the basis of MOI value, the lytic capability of these two phages was slightly higher than AHP42 or AKS96.

### General genomic feature

All four phages contained circularly permuted genomes of 45.7–46.8 kb (43.8–44 mol% G+C) encoding 74–81 open reading frames (ORFs) and 1 arginyl-tRNA (Tables 3 and S1−S4). Furthermore, 18−21 rho-dependent terminators and 5−10 promoters recognized by host RNA polymerase were identified. The majority (68–72 ORFs, 86–94%) of the proteins displayed homology to proteins of other T1-like phages with 32−39 of DNA replication, morphogenesis, genome packing and lysis (Tables 3 and S1−S4). Based on functional comparison of the ORFs to the NCBI database of non-redundant protein sequences, none of the genes encoded for proteins associated with pathogenesis, integration or antibiotic resistance.

**Table 3:** General genome features of AHP24, AHS24, AHP42 and AKS96.

	Phages			
Feature	AHP24	AHS24	AHP42	AKS96
Size (bp)	46,719	46,440	46,847	45,746
Sequence coverage	31.6	74.6	23.4	47.5
G+C content (%)	43.8	43.8	44	43.9
Total ORFs	78	81	76	74
Average ORF size (bp)	552	531	560	569
% of genome coding for proteins	92.2	92.8	90.9	92.2
No. of gene products similar to known proteins, total	73	71	72	69
No. of gene products similar to known T1, total	72	70	71	68
No. of conserved hypothetical proteins with unknown function	43	42	44	42
No. of hypothetical proteins	5	10	5	4
No. of tRNAs	1	1	1	1
No. of σ^70^ promoters	5	6	10	7
No. of rho-independent terminators	18	18	21	21

### Comparative analysis

**Figure 1:**
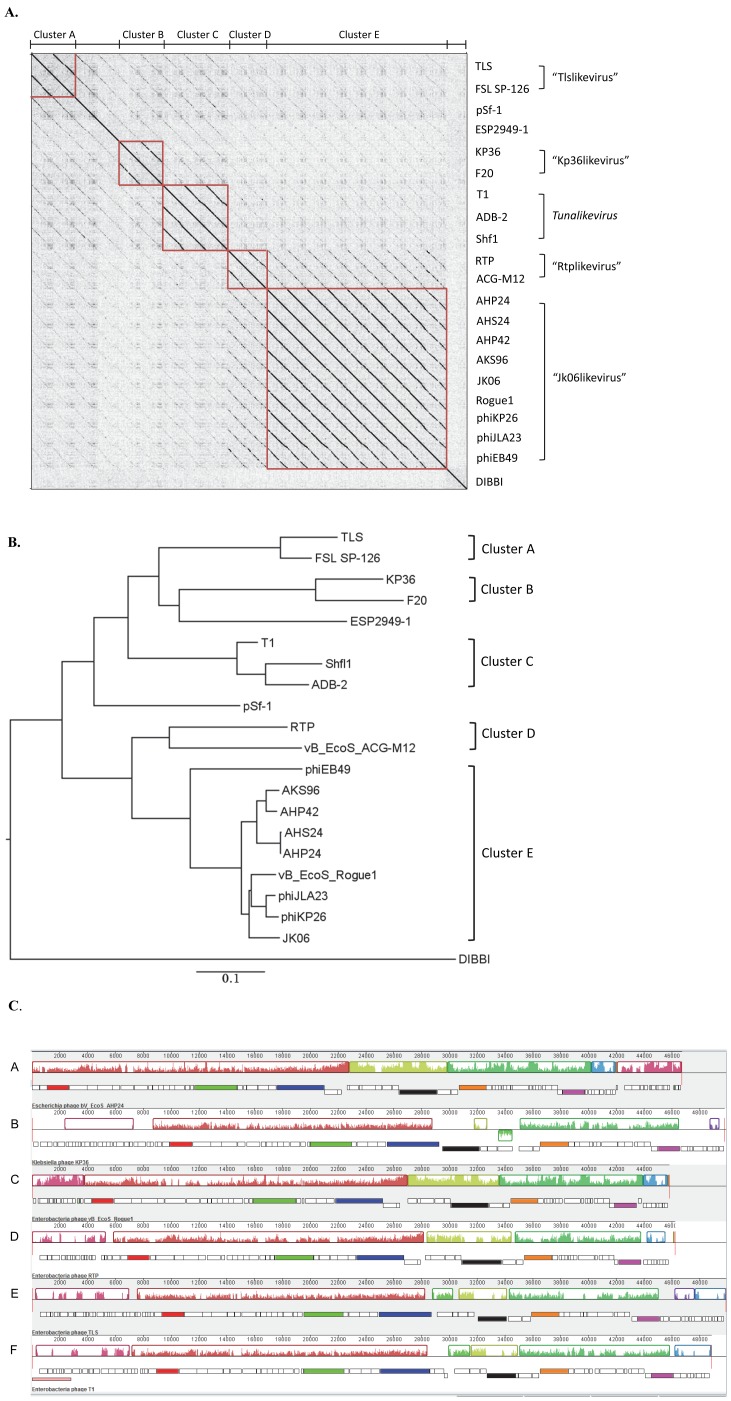
Comparative genomic analysis of the 21 known T1-like phages. A, Dot plot alignment of nucleotide identity of the 21 known T1-like phages using Gepard [61]. The vertical axis shows the phage IDs and horizontal axis indicates phage clusters (highlighted in red box). The apparent black diagonal lines indicate high degrees of nucleotide sequence identity; while each phage shows 100% identity to itself (displayed as diagonal line). B, Phylogenetic analysis of whole genomes of the 21 known T1-like phages by ClustalW algorithm. Scale bar represents 0.1 substitutions. C, Whole genome comparisons of phages AHP24 (A), KP36 (B), Rogue1 (C), RTP (D), TLS (E) and T1 (F) using a progressive MAUVE alignment [62]. The degree of sequence similarity is indicated by the intensity of the colored region. The contiguous black boxes under the colored region represent the position of the genes; red, large subunit of terminase; green, tail tape measure protein; blue, tail fiber protein I; black, tail fiber protein II; orange, helicase; pink, common hypothetical protein.

Comparative computational genomic analysis revealed that the four T1-like phages were collinear (>90.8% pairwise similarity), with AHP24 and AHS24 having the highest sequence identity (99.2%). All four phages were 80.8–86.5% identical to phages JK06, vB_EcoS_Rogue1, phiJLA23 and phiKP26 and 65% similar to phiEB49 in Cluster E and <56% related to other known T1-like phages (Fig. 1A, Table 4). At the protein level, computational analysis of CoreGenes demonstrated that compared to phage vB_EcoS_Rogue1, phages AHP24 and AHS24 shared greatest number of proteins (67–68, Avg. 91.2% similarity) in common, followed by AHP42, AKS96, phiKP26 and phiJLA23 (65, 87.8%), phiJLA23, JK06 and phiEB49 (56–58, Avg.77%)(Table 5).

**Table 4:** Pairwise nucleotide sequence identity of T1-like collinearized genomes as calculated by EMBOSS Stretcher[44], [45].

Cluster	Genus	DNA sequence similarity (%)							
		Within genus	between genus and/or species^a^						
			“Tlslikevirus”	“Kp36likevirus”	*Tunalikevirus*	“Rtplikevirus”	“Jk06likevirus”	Putative orphan species	
								pSf-1	ESP2949-1
A	“Tlslikevirus”	82.6	100	53.4	54.9	48.9	50.3	57.4	56.4
B	“Kp36likevirus”	72.8		100	53.3	48.6	49.3	52.6	53.3
C	*Tunalikevirus*	77.6–81.5			100	50.6	51.3	54.7	54.6
D	“Rtplikevirus”	65.1				100	55.7	49.6	49
E	“Jk06likevirus”	64.2–99.2					100	50.5	49.9

a DNA sequence similarity between different genera/or species was calculated using phage TLS a reference genome for the “Tlslikevirus”, phage KP36 for the “Kp36likevirus”, phage T1 for the *Tunalikevirus*, phage RTP for the “Rtplikevirus” and phage JK06 for the “Jk06likevirus”.

**Table 5:** Proteomic analysis of T1-like collinearized genomes using CoreGenes 3.0 program [46]–[48].

Cluster	Genus	No. of common proteins shared (%)^a^							
		Within genus	between genus and/or species						
			“Tlslikevirus”	“Kp36likevirus”	*Tunalikevirus*	“Rtplikevirus”	“Jk06likevirus”	Putative orphan species	
								pSf-1	ESP2949-1
A	“Tlslikevirus”	72 (82.8)	(100)	45 (51.7)	46 (52.9)	42 (48.3)	38 (43.7)	55 (63.2)	35 (46.7)
B	“Kp36likevirus”	67 (83.8)		(100)	41 (51.3)	38 (47.5)	36 (45)	42 (52.5)	38 (47.5)
C	*Tunalikevirus*	61–66 (78.1–84.6)			(100)	38 (48.7)	37 (47.4)	47 (60.3)	36 (46.2)
D	“Rtplikevirus”	58 (77.3)				(100)	51 (68)	42 (52.5)	38 (47.5)
E	“Jk06likevirus”	56–68 (75.7–91.2)					(100)	41 (55.4)	33 (44.6)

a Percentage value in each row is the ratio of homologs shared to total genes which was calculated using phage TLS a reference genome for the “Tlslikevirus”, phage KP36 for the “Kp36likevirus”, phage T1 for the *Tunalikevirus*, phage RTP for the “Rtplikevirus” and phage Rogue1 for the “Jk06likevirus”.

To obtain a global phylogenetic overview of the relationships between the T1-like phages, we employed genomic dot-plots of these genome sequences against each other (Fig. 1A). Clearly, nucleotide sequence aligned well within each cluster. Phylogenic analysis of whole genome also demonstrated that phages within each cluster shared close relatedness at nucleotide level (Fig. 1B). Nucleotide similarity of phages within each cluster was 82.6% (Cluster A), 72.8% (Cluster B), 77.6–81.5% (Cluster C), 65.1% (Cluster D) and 64.2–99.2% (Cluster E), whereas nucleotide identity shared between each cluster was 48.6–55.7% (Fig. 1C, Tables 2 and 4). Phages pSf-1 and ESP2949-1 demonstrated lower nucleotide similarity to phages from each genus (Table 4). Computational analysis of CoreGenes showed that phages within same cluster had greater number of homologues (75.7–91.2%) than those among different clusters (43.7–68%; Table 5). Orphan phage species pSf-1 and ESP2949-1 did not have over 55 gene products (<64%) in common as compared with other phages in each cluster. Considering the close relatedness at both nucleotide and protein level exhibited by the phages within each cluster, we propose the establishment of a new subfamily “Tunavirinae” which can be divided into five genera, i.e. “Tlslikevirus” (Cluster A), “Kp36likevirus” (Cluster B), *Tunalikevirus* (Cluster C), “Rtplikevirus” (Cluster D) and “Jk06likevirus” (Cluster E) (Fig. 1, Tables 4 and 5), each of which is named after the first isolated phage of its type.

**Figure 2:**
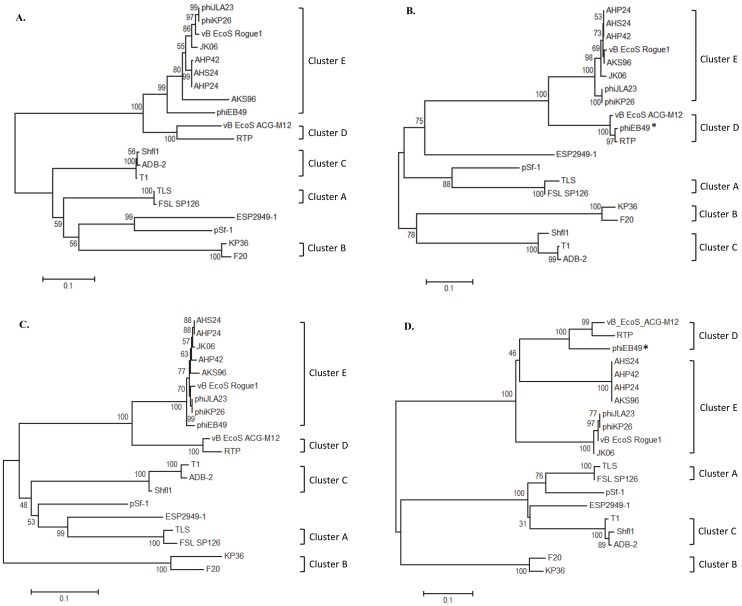
Evolutionary relationships of major proteins. The evolutionary history was inferred using the Neighbor-Joining method [63]. The optimal tree with the sum of branch length for A, large subunit of terminase ( = 2.05), B, portal protein ( = 2.2), C, tail fiber ( = 1.61), D, major capsid ( = 2.05), is shown; The percentage of replicate trees in which the associated taxa clustered together in the bootstrap test (500 replicates) are shown next to the branches [64]. The tree is drawn to scale, with branch lengths in the same units as those of the evolutionary distances used to infer the phylogenetic tree. The evolutionary distances were computed using the JTT matrix-based method [65] and are in the units of the number of amino acid substitutions per site. The analysis involved 20 amino acid sequences. All positions containing gaps and missing data were eliminated. There were a total of 429 positions (A), 244 positions (B), 807 positions (C), 305 positions (D), in each final dataset. Evolutionary analyses were conducted in MEGA5 [66]. Scale bar represents 0.1 substitutions. The asterisk represents phages of which evolutionary relationships of major proteins differed from those of whole genome.

Phylogenetic trees were constructed to further investigate common proteomic features for the large subunit of terminase (TerL), portal protein (PorT), tail fiber (FibA) and major capsid proteins (CapS) (Fig. 2). Overall, these analyses substantiated the establishment of the proposed genera. Interestingly, PorT and CapS of phage phiEB49 were more closely related to those from the “Rtplikevirus” (84.4–93% aa identity, ID) than those from the “Jk06likevirus” (70.4–75.2% aa ID). Within the “Jk06likevirus”, CapS from AHP24, AHS24, AHP42 and AKS96 (100% aa ID) was found to be 70.4–72.3% (aa) related to that from phages JK06, Rogue1, phiKP26 and phiJLA23 (97.8–99.7% aa ID). Likewise high diversity of the whole genome presented by orphan phages pSf-1 and ESP2949-1, low amino acid sequence similarities (<73.5%) were identified for each of the proteins studied, as compared to those of other members of the T1-like family.

### Proteomics

**Figure 3:**
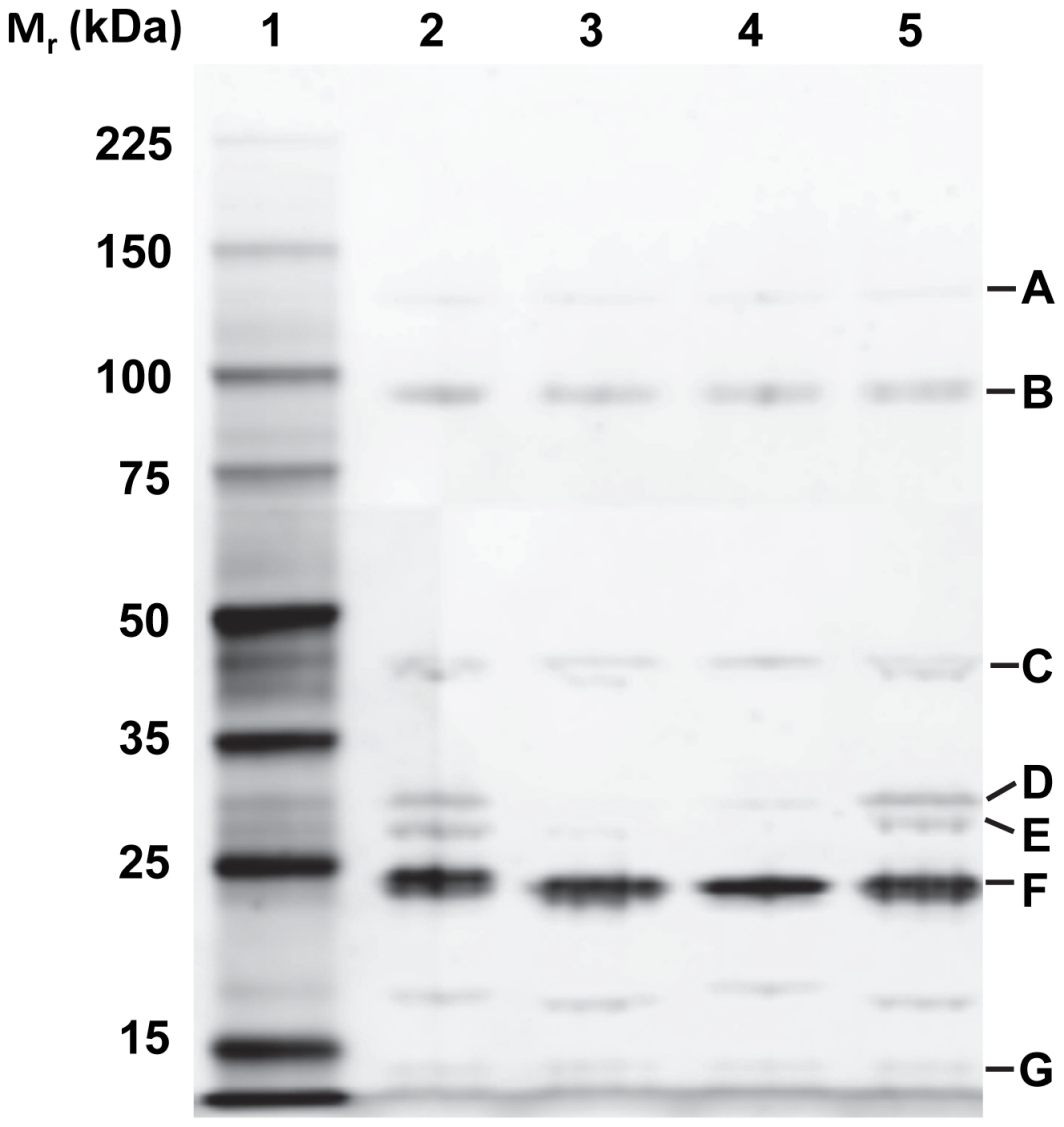
T1-like structural proteins (Lane 2-5) alongside the standard marker (Lane1) separated on 12% SDS-PAGE gel and visualized by Coomassie brilliant blue R250 stain. A, tail fiber protein; B, tail tape measure protein; C, portal protein; D, major capsid protein; E, minor tail protein; F, major tail protein; G, conserved hypothetical protein.

SDS-PAGE revealed that the structural proteins generated identical banding patterns among the four phages (Fig. 3). Further shotgun proteomics by liquid chromatography-tandem mass spectrometry identified up to 52% of the amino acids in seven structural proteins including tail fiber, tail tape measure protein, major capsid, portal protein as well as major and minor tail proteins (Table 6 and Fig. 3). A major capsid protein (Fig. 3, band D) was observed to have a molecular mass of 29.7 kDa, similar to the 33 kDa of the major head subunit P7 protein previously identified from T1 phage [38]. Also, a conserved hypothetical protein with a molecular mass of 14.2 kDa (Table 6; Fig. 3, band G) was similar to P11 (16 kDa) from phage T1, which has been proposed to be a second major head component that stabilizes the later stages of head assembly [39]. A major 23.6 kDa tail protein (Table 6; Fig. 3, band F) was consistent with a major tail protein from phage Rogue1 (gp29, 25.9kDa) [40] and from phage T1 (P10, 26 kDa) [39].

**Table 6:** Structural proteins of phages AHP24, AHS24, AHP42 and AKS96 identified by mass spectrometry.

Gel band	ORF in T1-like phages				Theoretical mass (kDa)	Observed mass (kDa)	Putative function	No. of peptides	Sequence coverage (%)
	AHP24	AHS24	AHP42	AKS96					
A	24	25	25	23	124.4	128	Tail fiber protein	11–23	11–24
B	18	19	19	18	108.9	94.8	Tail tape measure protein	43–55	43–50
C	04	04	04	04	46.3	43.9	Portal protein	14–25	29–47
D	09	10	10	09	34.7	29.7	Major capsid protein	3–7	9–27
E	21	22	21	20	28.1	27.7	Minor tail protein	3–10	10–50
F	15	16	16	15	23.5	23.6	Major tail protein	5–9	31–52
G	12	13	13	12	13.5	14.2	Conserved hypothetical protein	3–6	27–42

## Discussion

This study revealed that phages AHP24, AHS24, AHP42 and AKS96 are closely related members of new proposed genus–“Jk06likevirus”. Not surprisingly, the highest degree of nucleotide identity was shared between AHP24 and AHS24, as they were isolated simultaneously from fecal pats and manure slurry from the same feedlot pen [19]. AHP42 and AKS96 originated from different feedlots, but displayed the second highest degree of nucleotide sequence similarity, a result that confirms our previous findings of genomic relatedness of these two isolates based on restriction enzyme profiles [18]. Our ongoing work has also characterized a number of additional STEC O157:H7-infecting phages with TEM morphology, genome size and restriction enzyme profiles (Niu et al. unpublished data) that are similar to the four phages in this study, possibly because they were also obtained from the same commercial feedlots in 2007 [19]. This may suggest that “Jk06likevirus” are widespread in Alberta feedlots. All four phages were active against a broad range of STEC O157:H7 reference strains, but did not target non-O157 *E. coli*, suggesting that they could be used to control STEC O157:H7 without harming generic commensal *E. coli*. Also, the four phages exhibited strong lytic capability against vast majority of PT strains of STEC O157:H7, although lytic capability may vary with propagation hosts. This would make them effective biocontrol agents and possible low dosage required for therapeutic application.

A total of 21 phages with genome sizes ranging from 43 to 52 kb, similar genomic structure and TEM morphology have been described. Based on current taxonomic classification of ICTV, these phages were classified as T1-like phage (*Tunalikevirus*) within *Siphoviridae*. Undoubtedly, more T1-like phages will be identified in the future and there is a need to establish a more defined taxonomic system in order to explore the evolutionary relationships and genetic linkages in these types of phage. In the present study, we aligned whole genome sequences from all 21 T1-like phages using the ClustalW algorithm, which has been widely used for nucleotide sequence alignment of viruses [41]-[43]. The phylogenetic analysis showed that the T1-related phages fall into five clusters. Moreover, computational EMBOSS Stretcher [44], [45] and CoreGenes programs [46]–[48] showed that phages within each proposed genus were more closely related than those among genera at both the nucleotide and protein level. This was also confirmed by the phylogenetic analysis of four key functional phage proteins. The fact that the viruses related to JK06 have been isolated independently in Israel (JK06) (GenBank Assession #, NC_007291), Canada (vB_EcoS_Rogue1, AHP24, AHS24, AHP42, AKS96) [40] and Mexico (phiKP26, phiJLA23) [49], [50] between 2005 and 2011 indicates that these similar phages are widely distributed, and that horizontal gene transfer does not always prevent the characterization of bacteriophage evolution. Similar finding have been noted as part of the Phage Hunters Integrating Research and Education (PHIRE) program (http://phagesdb.org/) and for the global distribution of viruses related to *Listeria* phage A511. The results indicate that a new subfamily, the “Tunavirinae” created within the family *Siphoviridae* containing the following genera: a modified *Tunalikevirus* (T1, ADB-2, Shfl1) [51], [52], “Tlslikevirus” (TLS, FSL SP-126) [51], [53], “Kp36likevirus” (KP36, F20) [54], [55], “Rtplikevirus” (RTP, vB_EcoS_ACG-M12) [56], [57]; and “Jk06likevirus” (JK06, vB_EcoS_Rogue1, AHP24, AHS24, AHP42, AKS96, phiJLA23, phiKP26, phiEB49) [40], [49], [50], [58]. This would leave two putative orphan species: pSf-1 [59] and ESP2949-1 [60] to be further classified as more phages are characterized. There is a move within ICTV to eliminate the order *Caudovirales*, and its three families (*Myoviridae, Siphoviridae* and *Podoviridae*) as they are not compatible with emerging genomic and proteomic information on phage phylogeny.

Mitigation of STEC O157:H7 has been a challenge in feedlot cattle. The newly discovered four members of “Jk06likevirus” exhibited broad host range and strong lytic capability against STEC O157:H7, emphasizing efficacy and suitability for phage-based biocontrol of this zoonotic pathogen. In this study, we also proposed further classification of the 21 known T1-like phages into one subfamily with five genera, constructing a basis for proper identification of new phages within the same type. Genomic- and proteomic- based taxonomic classification of phages would facilitate a better understanding of phage diversity and genetic traits involved in phage evolution.

## Supporting Information

Table S1 Feature of phage AHP24 gene products and their functional assignments.
(XLSX)

Table S2 Feature of phage AHS24 gene products and their functional assignments.
(XLSX)

Table S3 Feature of phage AHP42 gene products and their functional assignments.
(XLSX)

Table S4 Feature of phage AKS96 gene products and their functional assignments.
(XLSX)
